# Evaluation of micro shear bonding strength of two universal dentin bondings to superficial dentin by self etch and etch-and-rinse strategies

**DOI:** 10.4317/jced.54740

**Published:** 2018-09-01

**Authors:** Pedram Daneshkazemi, Amir Ghasemi, Alireza Daneshkazemi, Fahime Shafiee

**Affiliations:** 1 Post graduate student, Department of orthodontics, Faculty of Dentistry, Islamic Azad University of Isfahan(khorasgan), Isfahan, Iran; 2 Associate Professor of Operative and esthetic Dentistry Department, Shahid Beheshti University of Medical Sciences, Tehran, Iran; 3 Associate Professor of Operative and Esthetic Dentistry Department, Social Determinant of oral health research center, Shahid Sadoughi University of Medical Sciences, Yazd, Iran; 4 Post graduate student, Department of operative and esthetic dentistry, Shahid Sadoughi University of Medical Sciences, Yazd, Iran

## Abstract

**Background:**

Universal bondings can be used either with the etch-and-rinse or self-etch technique. Thus, the present study was done to evaluate the micro-shear bonding strength of two types of Universal Bondings to superficial dentin i.e self-etch and etch and rinse.

**Material and Methods:**

The samples included 70 tooth blocks taken from 35 extracted sound premolar teeth. The superficial dentin was exposed to grinding by 800 grit silicon carbide Disk. The samples were randomly divided into 5 equal groups (14 samples in each group). Scotch bond universal (3M/USA) and All bond universal (BISCO/USA) were applied by self-etch and etch and rinse technique in group 1-4 and Adper Single bond 2 (3M/USA) was used in group 5 as etch and rinse for the control group. Z250 XT (3M/USA) resin composite was bonded in tygon tube on surfaces of samples and were cured. Specimens were stored in distilled water at 37ºC for 24 h and then subjected to the micro shear bond strength test in a universal testing machine at a crosshead speed of 0.5 mm/min. The data were analyzed by two-way ANOVA and Tukey test. Failure mode was determined using a stereomicroscope under 20X magnification. Significance level was considered 0/05.

**Results:**

The mean of micro-shear bonding strength and Standard Deviation of groups in Mega Pascal are respectively: 35.74 (6.21), 29.50 (3.89), 24.60 (3.53), 31.47 (4.73), 18.09 (3.87). The self-etch technique for Scotch bond Universal and the etch and rinse technique for All bond Universal showed higher micro shear bonding strength. Adper single bond 2 showed the lowest bond strength to a significant level in comparison to other groups (*p*<0.05). Failure mode was predominantly adhesive.

**Conclusions:**

The micro shear bonding strength of universal adhesives was highly bonding-dependent. Universal bondings had higher micro-shear bonding strength than Adper single bond 2.

** Key words:**Bond strength, dentin, self-etch, etch-and-rinse, universal bonding.

## Introduction

Nowadays, patients’ desire to have beautiful teeth and using tooth-colored restorations has increased, so that composites are used to repair both anterior and posterior teeth. Therefore, the need for proper bonding seems undeniable (1).

So far, different generations of dentin bonding agents have been developed and introduced to the market. Along with resin composites, these bondings can be an alternative for mineral materials of solid tissues of tooth and are attached to the teeth micromechanically (2).

The main purposes of producing new generations of dentin bonding agents include the applying their capability of attaching to the tooth, reducing the process, and simplifying their application. Typical bonding systems that are available in the markets are divided into two categories: self-etch and total etch. In total-etch systems, phosphoric acid is initially used at 35-37%; this is likely to cause etching and removing the smear layer as well as exposing collagen fibrils. In case of excessive drying of the tooth after the etching process, there is the likelihood of collapsing collagen fibers that reduce the strength of the bond to the tooth structure. In the early stages of the self-etch bonding systems, using phosphoric acid is not necessary. Therefore, in areas where access the isolation is hard, these systems can be used. However, they have low bond strength to the enamel. Over the recent years, new types of bondings have been produced, and given their application method and junction strategy, they are named universal or multipurpose (multi-mode ). They can act as both self-etch and total-etch, and have the capability to create appropriate bonding with wet or dry dentin. Moreover, according to the manufacturer’s information, they can be attached to enamel, dentin, porcelain, amalgam and metal (2,3).

Post-treatment paining sometimes felt by using other dentin bondings does not exist in this type of bonding. Universal bondings are suitable for bonding to all indirect surfaces, including zircon, alumina, glass-ceramics and metals. They have a reasonable marginal integrity, and they are in one single step and cured after application (2). Examples of these types of bonding include Scotch Bond Universal (3M) and All Bond Universal (Bisco).

Given the limited studies available on the Universal Bond, the recommendation for their wide application is provided with caution. In the study of Muñoz *et al.* about the features of immediate universal bond of adhesives, superficial dentin of occlusal surface of teeth was exposed. Three universal adhesives (all bond, scotch bond and peak bond) were used in two forms of self-etch and total etch technique. Moreover, micro hybrid composite (Opallis) was used. The results showed that the function of universal adhesives depends on the type of material (4). The results of Thanaratikul’s study showed that Adper single bond 2 had the lowest and Clearfil SE bond had the highest bond strength. Moreover, the bond strength of Scotch bond universal was similar in both self-etch and total etch methods (5). In the study of Michaud *et al.* about the Effect of universal adhesive etching modes on bond strength to dual-polymerizing composite resins, was shown a self-etch protocol provided significantly higher bond strength when Scotchbond Universal was used, whereas with All-Bond Universal, an etch-and-rinse protocol, provided higher bond strength (6).

According to contraindications found in other studies, the aim of this study was to evaluate the micro shear bond strength of two types of universal bonding agents to superficial dentin surface with two self-etch and total etch strategies. The following null hypotheses were tested in this study: (1) there would be no significant difference between The microshear bonding strength of the two types of universal bonding with each other and the control group; (2) there would be no significant difference in microshear bonding strength of scotch bond universal in self or total etch Techniques.

## Material and Methods

The samples included 70 tooth blocks taken from 35 extracted sound human premolar teeth. The samples were randomly divided into five 14-tooth groups (n=14). considering the significant level of 5% and test power of 80% and according to maximum standard deviation of 2.3 to achieve a significant difference of 2.5 in the mean of groups, 14 samples per group were needed.

Teeth were stored in normal saline solution. For the infection control, the teeth were immersed in a 0.5% T solution of chloramine for 24 hours before conducting the study. The root of the teeth was cut off and the enamel of the occlusal surface was removed by the disk with 0.3 mm thickness under cooling by water spray with sectioning Device (GH-5 / Hamco machines NC. / Rochester / New York / USA) in order to make flat ground superficial dentin. Then, from each tooth, two sections with a thickness of 1.5 mm were created, and a total of 70 samples were obtained. The upper surface of the samples were ground by silicon papers with 400-600-800 grit of coarseness respectively. Then the samples were placed in distilled water for 14 days.

Group 1: Scotch Bond Universal (3M / USA) using Self-etch strategy, Z250 XT resin Composite (3 M / USA)

Group 2: Scotch Bond Universal by Total etch strategy and Z250 XT resin Composite.

Group 3: All Bond Universal (Bisco / USA) by self-etch strategy and Z250 XT resin composite.

Group 4: All Bond Universal by Total etching strategy and Z250 XT resin composite.

Group 5: Control group that Adper Single Bond II (3M/USA) by total etch technique and z250 XT resin composite was used.

Additionally, the features of the Dentin bondings used in this study are as follows .

Scotch bond Universal contains 10-MDP, Dimethacrylate resins, silane, initiator, filler, polyacrylic acid, copolymer, HEMA, ethanol, water, phosphoric acid, and it’s PH is 2.7.

All bond Universal contains 10-MDP, Dimethacrylate resins, HEMA, Ethanol, water, initiator, and it ‘s PH is 3.2.

Adper Single bond II contains Bis-GMA, Dimethacrylate resins, photoinitiator, HEMA, copolymer, filler, ethanol, water, 10% by weight of 5 nm-diameter spherical silica particles, and it’s PH is 4.3 (3).

The procedure in group 1 was that at first the specimens were washed and dried, and the drying was done to the extent to remove moisture. Subsequently, using a microbrush in the middle of the prepared surface, two layers of Scotch Bond Universal was used according to the manufacturer’s instructions, and they were cured for 10 seconds using the Optilux 501 (kerr Corp. USA) halogenic light curing device. Then, the Tigon tubes were placed on the bonded region and the Z250 XT resin composite (with a filler volume of 68% vol. And 82% by weight) (7) was packed to the bonded site and cured for 20 seconds. It should be noted that the light intensity of the device was investigated in several steps by the Demetron (kerr Corp.USA) light meter to ensure the light stability of the device during the study. Moreover, at the exposure stages, the distance between the tip of the light cure device and the surface of the specimens was minimal. As for group 2, the procedure was similar to that of group 1, but the application of Scotch Bond Universal was performed using total etch strategy, and acid etching is used by scotch bond etchant (3M ESPE / USA)(35% phosphoric acid) for 15 seconds. The etching surface of the specimens was washed and excess water was removed by Using cotton, as the surface of the dentin surface was kept moist and the remaining steps were similar to those of Group 1. The procedure in group 3 was similar to that of group 1, but dentin bonding agent was All-bond universal used with self-etch strategy. The procedure of Group 4 was similar to that of Group 2, but dentin bonding agent was all-bond universal that was used with total-etch strategy. In group 5 (control group), the procedure was similar to that of group 2. However, it was different only in terms of using Adper Single Bond 2, followed by using Z250 XT resin composite and conducting the curing step.

Specimens were stored in distilled water at 37ºC and 90% humidity for 24 hours and then subjected to the micro shear bond strength test in a universal testing machine (Bisco / USA) at a crosshead speed of 0.5 mm/min. The failure force was recorded in blank tables and converted into Mega Pascal unit according to the dimensions of the bonded area. Moreover, mode of failure was investigated by using two examiner through observing the debonded surface levels separately by a stereomicroscope JB7701 (ZTEW / China) at × 20 magnification. At the end, the type of failure: Cohesive (failure exclusive within dentine or resin composite), adhesive (failure at resin/dentine interface), or mixed (failure at resin/dentine interface, which included cohesive failure of the neighboring substrates) was identified.

Data were analyzed by using two way ANOVA, one way ANOVA and Tukey HSD statistics exam.

## Results

The aim of this study was to investigate the micro shear bond strength of two types of universal bondings to dentin bonded area with self-etch and total etching strategy with Z250 XT resin composite. This study showed that there is a difference between the investigated groups in terms of micro shear bond strength. The highest average bond strength of scotch bond universal was related to self-etch bond strategy and the lowest micro shear bond strength belonged to the control group using single bond II, and the statistical difference between the two groups was significant (*P* <0.05) ( Table 1, Table 2).

**Table 1:**
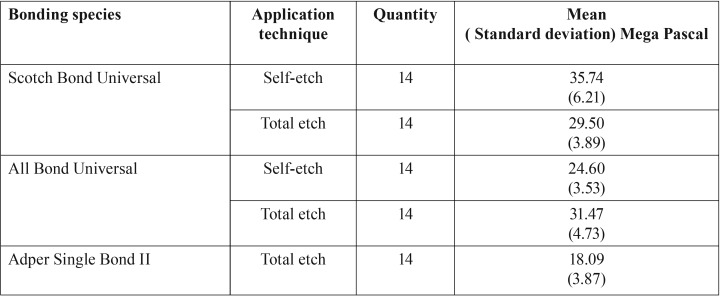
Micro shear bond strength values to superficial dentin in experimental groups.

**Table 2:**
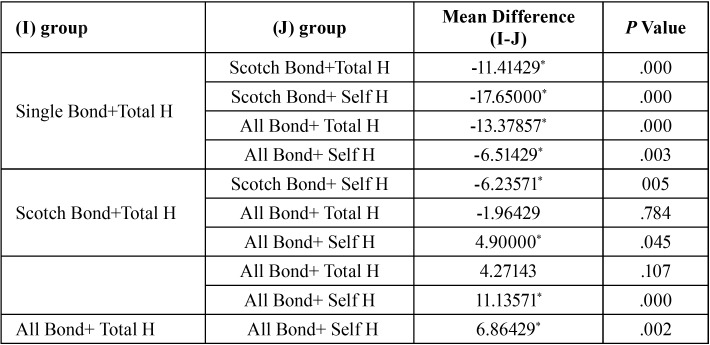
The effect of multiple comparisons between studied groups.

ANOVA one way statistic test showed that there is a significant difference between at least 2 groups of 5 studied groups (application technique) (0/0001> *p*). Scotch bond universal with self-etch strategy had the highest micro shear bond strength among the groups. The Tukey HSD test showed that there was no significant difference in terms of the micro shear bond strength between scotch bond universal group with total etching strategy and All bond universal with total etch strategy (*p* = 0.784). There was no significant difference between the scotch bond universal group with Total-etch strategy with All bond universal group with Self-etch strategy in terms of the micro shear bond strength (*p* = 0.45).There was no significant difference between scotch bond universal group with self-etch strategy and All bond universal group using total etch method in terms of micro-shear bond strength (*p*=0/107).

However, Tukey HSD statistical test, demonstrated that the difference between other groups was significant ( Table 2). In addition, both of universal bonding groups had higher bonding strength than that of the control group (*p*<0/05).

 Furthermore, Two Way ANOVA statistical test showed that bonding type effect was significant (*p*=0/01). Although effect of acid etching was not significant, the interaction between acid etching and bonding type appeared significant (*p*< 0.0001).

By examining samples under a stereomicroscope at × 20 magnification, the failure mode of each group was identified. The highest adhesive failure rate was observed in Adper single bond2, and the failure rate in other groups was between 70-80 %. As for the cohesive failure, Adper single bond2 had the lowest rate, and other groups had a range of 5-15% in terms of failure. Moreover, the rate of failure of Cohesive/ Adhesive, or mix type ranged 5-15% ( Table 3). Also, Statistical comparison between the groups was performed ( Table 4).

**Table 3:**
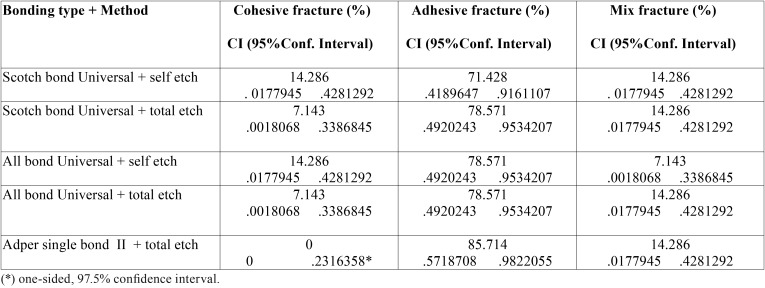
Fracture mode in experimental groups.

**Table 4:**
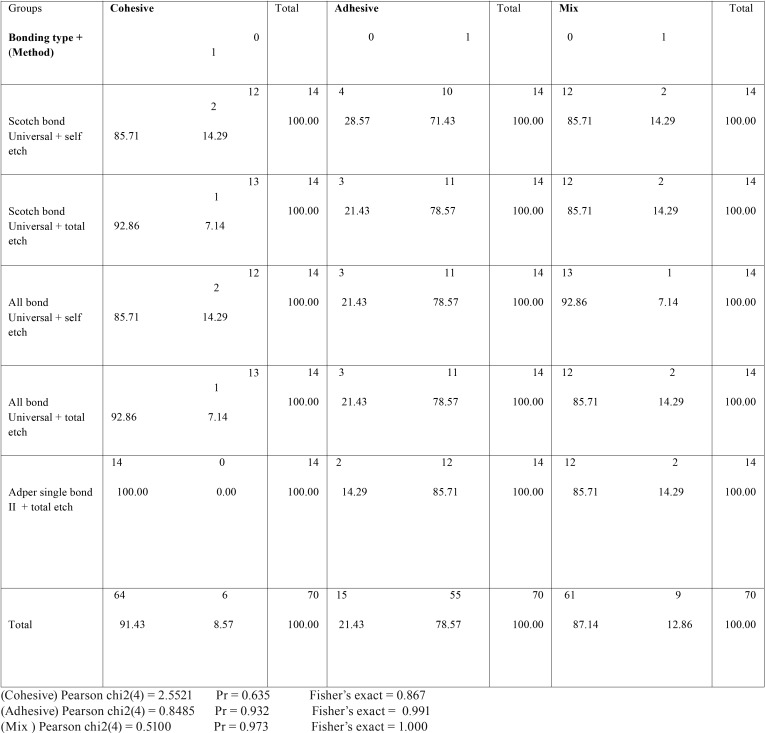
Statistical comparison between the groups.

## Discussion

Null hypotheses of this study were rejected. This study aimed to investigate micro shear bond strength of two universal bonding agents on superficial dentin surface by using two strategies- self-etch and total etch- and compare them with one another and also with a fifth generation bonding (etch-and-rinse) as the control group. Universal bondings used in this study include: Scotch bond Universal and All bond Universal as two experimental groups and also Adper single bond2 as the control group.

Our findings demonstrated that Scotch bond Universal using self-etch strategy has the highest micro shear bond strength, and single bond II had the lowest micro shear bond strength. Moreover, these results were consistent with those of the study conducted by Thanartikul (5) in which the micro shear bond strength of Scotch bond Universal on 40 deciduous incisor teeth was compared, using self-etch and total-etch strategies with Single bond II and Clearfil SE bond. Moreover, In Thanartikul ‘s study, Single bond2 had the lowest, and Clearfil SE bond and Scotch bond Universal using self-etch strategy, respectively had the highest connection to deciduous incisor dentin. In addition, Scotch bond using self-etch strategy had significantly more micro shear bond strength than that of the control group.

On the other hand, the results of current study are not consistent with those of the study conducted by Wagner *et al.* (8) and Martinez *et al.* (9). In the aforementioned studies, Scotch bond Universal using total-etch had more micro shear bond strength than self-etch strategy. Probable reasons for current results and the aforementioned studies could be the difference in resin composite type used, or the method used for teeth restoration in these different studies.

Moreover, PH bonding in Scotch Bond Universal is equal to 2/7 (3), which is due to existence of polyacrylic acid in its structure ( Table 1)causing better cohesion of this bonding with surface dentin ( the reason for higher strength in this type of bonding is using self-etch method, in comparison to self-etch bonding in All Bond Universal). Due to its higher acidity of monomers, using this bonding with self-etch method, makes higher micro shear bond strength for us, and makes us needless to use separate acid etch in advance. Bonding ability of universal adhesives to surface dentin has progressed significantly, but the cohesion to enamel is still unsatisfactory. So, doing separate acid etching on enamel is suggested before self-etch adhesives (10) (especially when using Adhesives with mild PH). Anyway, given the weakening bond strength, it is of high risk to use pre-etching inadvertently on surface dentin (11).

In our study, in comparison to control group (Adper single bond II) micro shear bond strength of universal dentin bondings to superficial dentin was higher and significantly different (*p*<0/0001). Moreover, the results of the present study are similar to those of the studies conducted by Thanaratikul *et al.* (5), Muñoz *et al.* (12), Kumari *et al.* (13) Muñoz *et al.* (4). Micro shear bond strength of universal bondings was less than that of the control group. The results of this study were similar to those of Bahrololumi’s study (3) that showed the water absorption in universal bondings is less when compared with Adper single bond II. This means progress in universal Adhesives when compared to fifth generation bondings.

In this study, when compared to self-etch strategy, All bond Universal with total-etch method had more micro shear bond strength on superficial dentin, and this difference was statistically significant (*p*=0/002). Although this was similar to results of Muñoz *et al.* (12), and Martinez *et al.* (9), it was inconsistent with the finding of the study conducted by Wagner *et al.* (8) in which shear bond strength with self-etch in all bond Universal was considered higher. PH in All bond Universal is 3/2 (3). Since this amount of acidity is not enough, using acid etch before its usage makes the bond stronger.

In those studies on superficial dentin, one element that may affect the bond strength is the method of preparing superficial dentin surface. Moreover, bond placement and method of attaching the composite to the surface are considered important. In the present study, flat ground superficial dentin was exposed in occlusal surface area by the disk. In the study of Martinez *et al.* (9) they used superficial dentin on occlusal surface but in the study conducted by Wagner et al. (8), deep occlusal dentin was created, and in the study conducted by Thanaratikul *et al.* (5), superficial dentin of buccal surface was created. As dentin characteristics on superficial and deep regions are different in occlusal or buccal and lingual surfaces, this is likely to affect bond strength in different surfaces or variable studies.

Another effective factor on bond strength is the composite type used on dentin surface. In this study, Z250 XT resin composite was used which is a kind of Nano hybrid composite for restoration of the anterior and posterior teeth. The size of this type of composite filler is equal to 20 nm. Amount of filler used in this composite is 68% volumetric and 82% weighing of silica (7). This type of composite was similar to the composite used in the study conducted by Marchesi *et al.* (15) study. However, in their studies, Muñoz *et al.* (12) and Thanartikul *et al.* (5) used Z350 resin composite, which is a type of Nano hybrid composite. Moreover, in their study, Wagner *et al.* (8) used Grandio resin composite that has 87% filler. In addition, in the study by Muñoz *et al.* (4) study, opallis resin composite was used, that is a kind of Nano hybrid composite with filler particles of 0/05 nm . Using different types of composites may also affect the results. The reason for using Z250 XT in this study was that test was conducted on posterior teeth, using a Nano hybrid composite seemed more suitable (7).

The method of using resin composite on dentin is also one of the most important factors in bond strength studies. Choosing and manipulating resin composite in various studies might be different and depends on the type of bond strength evaluated. In this study, Tygon tube was used to place the composite on the surface of the bonded area. For more precision, the acid etching and bonding process was limited to the Tygon range, as this could indicate the incorrect values of micro shear bond strength. Composite bonding method was consistent with the method conducted by Thanaratikul *et al.* (5) and Beltrami *et al.* (14) studies, where tygon and packing of resin composite was used in both studies. However, Martinez *et al.* (9), Muñoz *et al.* (12), Marchesi *et al.* (15) and Wagner *et al.* (8) have conducted their studies with build-up method using 4mm resin composite as incremental method in two layers. Because using resin composite in micro shear testing and micro tensile bond strength is different.

In the current study, the teeth were human premolar teeth that were extracted due to orthodontic treatments. Different teeth have been used in various studies, for example, in the study conducted by Martinez *et al.* (9), Wagner *et al.* (8), and Muñoz *et al.* (12) third molar teeth were used. Moreover, in the study conducted by Beltrami *et al.* (14) bovine mandibular incisors were used. In the studies of Thanaratikul *et al.* (5), decideous incisors were used,. Finally, in a study by Kumari *et al.* (13) human premolar teeth were used.

In this study, premolar teeth premolar teeth were collected for three months and they were then examined; this was similar to the procedure adopted in the studies conducted by Wagner *et al.* (8) and Thanaratikul *et al.* (5). However, Martinez *et al.* (9) and Muñoz *et al.* (4) performed the test in six months after tooth extraction. Obviously, the time span may also affect dentin specimens and cause some differences in results.

It should be noted that in the present study, the rate of force applied was 0.5 mm / min, which was consistent with that of the studies conducted by Wagner *et al.* (8), Muñoz *et al.* (4). However, in other studies, the speed of micro shear bonding force applied was different from that of the aforementioned studies. For example, in the study conducted by Thanaratikul *et al.* (5) the force applied was 1 mm per minute. The difference in the rate of force applied may also affect the difference in the results.

In this study, defect patterns in most of the groups were found to be adhesive defects. Overall, both adhesive and mixed fracture rates were significantly higher than cohesive failures in resin composite, which could be attributed to the use of All bond universal adhesive and Scotch bond universal adhesive. Moreover, Single bond II adhesive does not change the results of failure mode (16).These results were similar to those of the studies conducted by De Munck *et al.* (17) Martinez *et al.* (9) and Muñoz *et al.* (4). The results of the study conducted by Firat *et al.* (18) were inconsistent with those of our study. The absence of Cohesive failure in Adper Single Bond II group showed the minimum bond strength. The high strength of resin composite used in this study (Z250 XT), due to the size and amount of its fillers suggests an implicit confirmation of the weakening pattern in the weaker bonding in this study.

Some of the limitations of this study are the number of cut pieces in each group as well as the impossibility of measuring the strength of the micro shear bond of samples after a long time storage or aging.

## Conclusions

1. Due to the limitations of this study, Scotch Bond Universal with self-etch strategy had the highest micro shear bond strength.

2. All Bond Universal bonding by total-etch strategy had better micro shear bond strength than that of self-etch method.

3. Both All bond Universal and Scotch bond Universal had a significantly higher micro shear bond strength than Adper Single Bond II (*p*>0.05).

4. Failure mode of fracture in the experimental samples showed that the highest failure rates were in the form of Adhesive. Furthermore, the samples in the Adper Single Bond II group had the highest adhesive fracture rate.
